# Current Understanding of Circulating Biomarkers in Pulmonary Hypertension Due to Left Heart Disease

**DOI:** 10.3389/fmed.2020.570016

**Published:** 2020-10-07

**Authors:** Noah Todd, Yen-Chun Lai

**Affiliations:** ^1^Division of Pulmonary, Critical Care, Sleep and Occupational Medicine, Department of Medicine, Indiana University School of Medicine, Indianapolis, IN, United States; ^2^Department of Anatomy, Cell Biology and Physiology, Indiana University School of Medicine, Indianapolis, IN, United States

**Keywords:** pulmonary hypertension, group 2 PH, PH-HFpEF, HFpEF - heart failure with preserved ejection fraction, biomarkers

## Abstract

Pulmonary hypertension due to left heart disease (PH-LHD; Group 2), especially in the setting of heart failure with preserved ejection fraction (HFpEF), is the most frequent cause of PH. Despite its prevalence, no effective therapies for PH-LHD are available at present. This is largely due to the lack of a concise definition for hemodynamic phenotyping, existence of significant gaps in the understanding of the underlying pathology and the impact of associated comorbidities, as well as the absence of specific biomarkers that can aid in the early diagnosis and management of this challenging syndrome. Currently, B-type natriuretic peptide (BNP) and N-terminal proBNP (NT-proBNP) are guideline-recommended biomarkers for the diagnosis and prognosis of heart failure (HF) and PH. Endothelin-1 (ET-1), vascular endothelial growth factor-D (VEGF-D), and microRNA-206 have also been recently identified as new potential circulating biomarkers for patients with PH-LHD. In this review, we aim to present the current state of knowledge of circulating biomarkers that can be used to guide future research toward diagnosis, refine specific patient phenotype, and develop therapeutic approaches for PH-LHD, with a particular focus on PH-HFpEF. Potential circulating biomarkers identified in pre-clinical models of PH-LHD are also summarized here.

## Introduction

**Figure 1:**
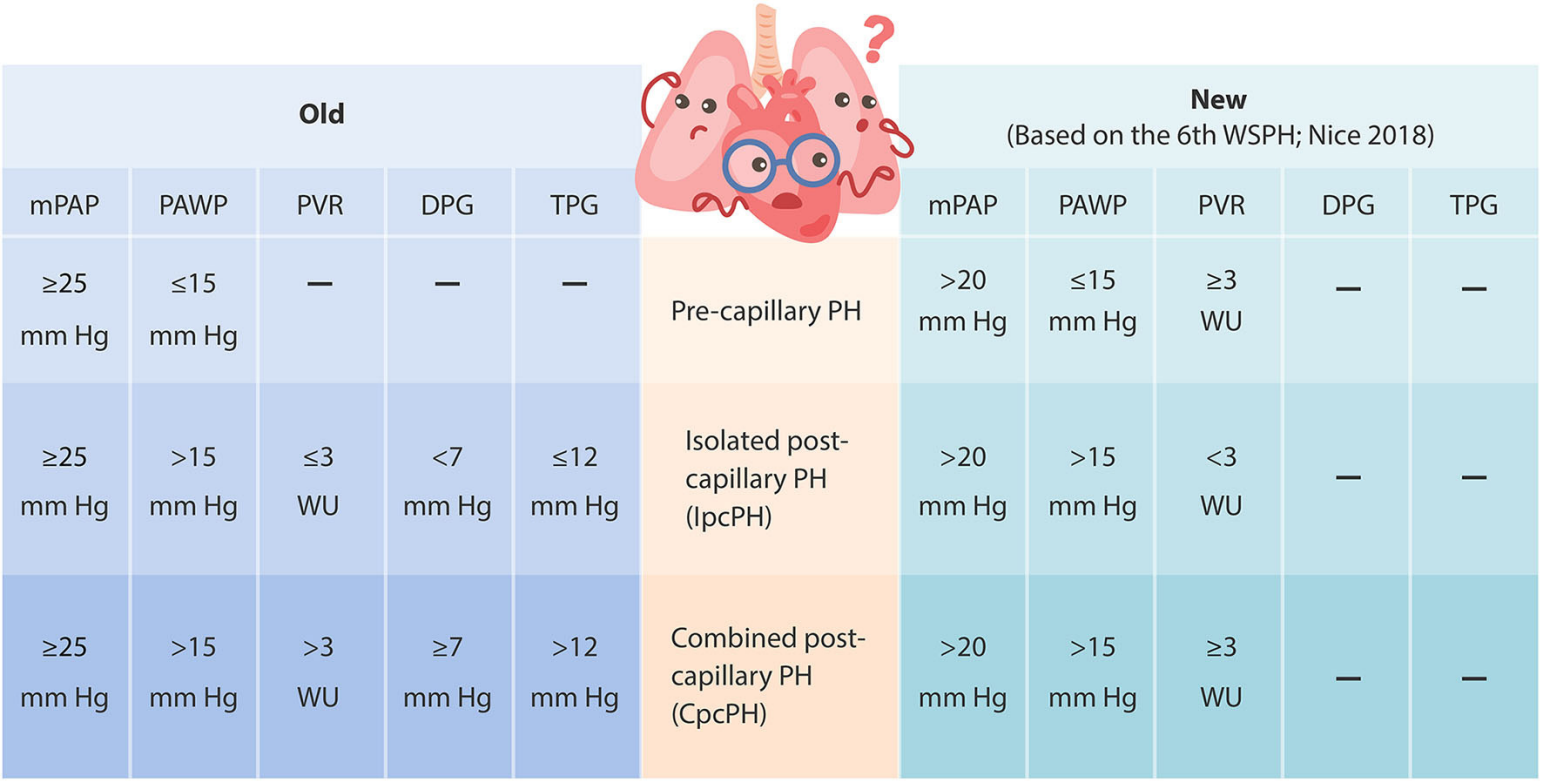
Updated hemodynamic definition and clinical classification of pulmonary hypertension (based on the 6th World Symposium on PH, Nice 2018). mPAP, mean pulmonary artery pressure; PAWP, pulmonary artery wedge pressure; TPG, transpulmonary gradient (defined as mPAP–PAWP); PVR, pulmonary vascular resistance (defined as TPG/cardiac output); DPG, diastolic pressure gradient (defined as diastolic PAP–PAWP).

Pulmonary hypertension due to left heart disease (PH-LHD, Group 2) is the most common cause of PH and is a growing public health problem with high morbidity and mortality (1–3). In fact, the presence of PH in patients with LHD has been associated with up to 5.6 times higher mortality compared to patients without PH (4). Left ventricular (LV) systolic dysfunction and diastolic dysfunction, left-sided valvular disease (aortic and mitral valve disease), and metabolic dysregulation are all known contributing factors that lead to increased LV filling pressure and the subsequent development of PH. Among these, PH attributed to LV diastolic dysfunction, also referred to as PH associated with heart failure with preserved ejection fraction (PH-HFpEF), is the most common form, although the reported prevalence varies from 23 to 83% due to variable definitions and diagnostic methods used to date (5–7). In addition to the already existing ambiguity in definition of this challenging syndrome, the 6th World Symposium on PH (Nice, 2018) has recently redefined PH-LHD as consisting of a mean pulmonary artery pressure (mPAP) greater than 20 mm Hg (revised from at least 25 mm Hg) and a pulmonary artery wedge pressure (PAWP) greater than 15 mm Hg (Figure 1) (8–11). Although this change reflects recent reports describing increased risk of disease progression in patients with a mild elevation in mPAP (21 to 24 mm Hg), this revision may lead to further confusion among clinicians when trying to diagnose PH in patients (9, 12).

Abnormally elevated LV filling pressures in HFpEF, heart failure with reduced ejection fraction (HFrEF, systolic heart failure), and valvular disease can all lead to elevated left atrium (LA) pressure, increased LA volume, and reduced LA compliance (5, 13). Basic cellular mechanisms affecting LV and LA remodeling include myocyte hypertrophy, up-regulated myocardial brain/B-type natriuretic peptide (BNP) expression in the failing ventricular and atrial myocardium in response to pressure increases and/or volume overload, endothelial dysfunction, vascular oxidative stress, inflammation, interstitial fibrosis, metabolic abnormalities, etc. (14–18). Increased LA pressure can then back up into the pulmonary circulation, leading to increased pulmonary venous pressure, which is in turn transferred to pulmonary capillaries, causing damage to the alveolar-capillary barrier (also known as alveolar-capillary stress failure). As the disease progresses, structural and functional changes regulated by chronic elevation in capillary pressure may trigger pulmonary vasoconstriction, reduce nitric oxide (NO) bioavailability, increase endothelin-1 (ET-1) production, and promote remodeling in the pulmonary arteries and veins, with various combinations of intimal proliferation, medial hypertrophy, and adventitial thickening (13, 17, 19–22). These pulmonary vascular abnormalities may then lead to a further increase in mPAP in addition to PAWP elevation, resulting in elevated right ventricular (RV) afterload and ultimately causing right-side heart failure. Depending on the extent of progressive pulmonary vascular abnormalities (also known as the pre-capillary component) to the underlying LHD, the backward transmission of elevated filling pressure can lead to an increase in pulmonary artery pressure proportionally (1:1 ratio) or disproportionately (> 1: 1 ratio) (17, 23–25). Patients with no significant pulmonary vasoconstriction or intrinsic pulmonary vasculopathy often exhibit proportional PH (isolated post-capillary PH, IpcPH). On the other hand, if chronically elevated LV filling pressure triggers pulmonary vasoconstriction and pathological pre-capillary remodeling to the point of having a high transpulmonary gradient (TPG, defined as mPAP–PAWP that exceeds 12 mmHg), elevated pulmonary vascular resistance (PVR, defined as TPG/cardiac output that exceeds 3 Wood units), and/or a high diastolic pulmonary gradient (DPG, defined as diastolic PAP–PAWP that equals or exceeds 7 mm Hg), patients exhibit out-of-proportion PH (combined pre-capillary and post-capillary PH, CpcPH). The severity of pre-capillary involvement can be established by the measured TPG, PVR, and/or DPG during right heart catheterization. In the past, TPG ≤ 12 mm Hg, PVR ≤ 3 Wood units, and/or DPG <7 mm Hg suggested IpcPH, and when elevated (TPG > 12 mm Hg, PVR > 3 Wood units, and/or DPG ≥ 7 mm Hg), suggested CpcPH (8). These features have been associated with mortality and cardiac hospitalizations in patients with CpcPH associated with HFpEF (CpcPH-HFpEF) (26, 27). However, the new definition from the 6th World Symposium on PH only includes PVR of less than 3 Wood units for IpcPH and PVR of at least 3 Wood units for CpcPH (Figure 1) (9–11).

Despite its significant prevalence, along with high morbidity and mortality, there is no Food and Drug Administration (FDA)-approved treatment for PH-LHD at present. While the use of pulmonary vasodilators has been proven to be effective in the treatment of pulmonary arterial hypertension (PAH; Group 1), the use of these agents in patients with PH-LHD has been shown to be ineffective or even harmful (see Fernandez et al. and Vachiéry et al. for recent reviews in detail) (22, 28). This is at least in part due to the lack of a concise and uniform definition for hemodynamic phenotyping, poor understanding of the underlying pathology and the impact of associated comorbidities, as well as the absence of specific biomarkers that can aid in the early diagnosis and management of this heterogeneous disease. Currently, BNP and N-terminal proBNP (NT-proBNP) are guideline-recommended biomarkers for the diagnosis and prognosis of HF and PH (29–31). However, these are not specific for PH-LHD and vary significantly with age, sex, body mass index (BMI), and renal function (30, 31). Several biomarkers have also been shown to be complimentary to the established natriuretic peptides in guiding disease management and have proven to be diagnostically valuable in distinguishing between IpcPH and CpcPH. In this review, we aim to provide an updated overview of the current state of knowledge regarding circulating biomarkers that may be used to guide future research toward diagnosis, refinement of specific patient phenotypes and development of therapeutic approaches for PH-LHD, with a particular focus on PH-HFpEF. We will first focus on the well-established natriuretic peptides, then we will review known biomarkers proposed for PAH and their new roles in PH-LHD. Finally, we will highlight some of the emerging circulating biomarkers, including those identified recently in pre-clinical models of PH-LHD.

## Well-Established Biomarkers: BNP and NT-proBNP

As LV dysfunction progresses, diastolic wall stress is the primary stimulus for myocardial BNP expression, which plays an important role in the regulation of cardiac remodeling, blood pressure, and intravascular volume (14–16). BNP is transcribed and produced primarily in the cardiomyocytes of the ventricles as prohormone proBNP, which is then cleaved into BNP (biologically active with a half-life of 20 min) and its N-terminal fragment NT-proBNP (biologically inactive with a half-life of 70 min). The precursor, BNP, and NT-proBNP are secreted directly into circulation in response to increased myocardial stretch mediated by pressure or volume overload (14–16).

### BNP/NT-proBNP in HFpEF and HFrEF

Plasma levels of BNP and NT-proBNP are elevated in patients with HF and increase in proportion to the degree of LV dysfunction and the severity of symptoms of HFpEF, HFrEF, and valvular disease (15, 32, 33). In fact, BNP and NT-proBNP are among the first circulating biomarkers included in the current guidelines for the diagnosis and risk stratification of HF (8, 31). While definitive cutoffs of BNP and NT-proBNP are not well-established for HFpEF or HFrEF (*see* Ponikowski et al. and Pieske et al. for the most recent recommended values) (18, 30), and various studies have used different thresholds, it is generally recognized that their levels are, on average, lower in HFpEF compared to HFrEF (30, 33). This may be due to the high prevalence of obesity in HFpEF populations, as disproportionately low BNP and NT-proBNP levels have been reported in obese patients, which may be related to the mechanisms involving natriuretic peptide degradation in adipose tissue, insulin resistance, and enhanced pericardial restraint (32, 34–37). Adipocytes strongly express the natriuretic peptide receptors NPR-A and NPR-C. As BNP binds to both NPR-A and NPR-C, it has been suggested that increased adipose tissue can lead to more efficient clearance of BNP in obese patients. However, lower plasma NT-proBNP levels seen in patients with obesity-related HF cannot be explained by the same mechanism because NT-proBNP does not bind to NPR-A or NPR-C (38, 39). Additionally, Obokata et al. found that patients with obesity-related HFpEF display more concentric LV remodeling, greater epicardial fat thickness, higher PAWP, RV dilation and dysfunction, elevated right atrial (RA) pressure, and increased total heart volume accompanied by lower NT-proBNP levels compared to non-obese HFpEF patients (32). In this study, PAWP was higher for any given NT-proBNP level in patients with obesity-related HFpEF compared to non-obese HFpEF patients; however, the relationship between NT-proBNP and LV transmural pressure, defined as intracavitary pressure (PAWP) minus the external pressure applied to the LV from pericardium and the right side of the heart (RA pressure), did not change in both obese and non-obese patients with HFpEF (32). As increased epicardial fat and higher heart volume exacerbate pericardial restraint, which may reduce wall stress as external pressure applied to the ventricle increases, these data may provide an alternative mechanism for the lower concentration of NT-proBNP observed in patients with obesity-related HFpEF (32, 37). Nevertheless, even mild elevations in NT-proBNP have been found to correlate with an increased risk of HF among individuals with obesity (40).

### BNP/NT-proBNP in PH-HFpEF and PH-HFrEF

Using a BNP cutoff value of > 100 pg/ml, an analysis from the Northwestern University HFpEF Program reported that up to 70% of patients with confirmed HFpEF have elevated BNP, which is associated with significantly higher mPAP, RA pressure, and PVR, along with higher rates of HF hospitalization, cardiovascular hospitalization, and death compared to HFpEF patients with “normal” BNP (≤ 100 pg/ml) (41). Elevated BNP levels were also found to be associated with an increase in PA systolic pressure (PASP) in PH patients associated with HFpEF, HFrEF, or valvular disease (42). Significantly higher BNP levels were observed in patients with CpcPH (50% of whom have LV ejection fraction <50%) compared to that of patients with PAH (25). Similarly, CpcPH-HFpEF patients were found to have about 1.8-fold higher levels of NT-proBNP compared to IpcPH-HFpEF patients, which was associated with an increased frequency of HF hospitalizations, reduced RV-vascular coupling (as measured by the ratio between tricuspid valve annular plane systolic excursion and PASP; TAPSE/PASP), impaired RV function, and severely depressed exercise capacity (43). Hussain et al. reported that NT-proBNP values were overall higher in HFpEF patients with PH, as compared to patients without PH. NT-proBNP levels were also higher in HFpEF patients with RV dysfunction (decreased TAPSE) despite the absence of PH. A moderate negative correlation between NT-proBNP and TAPSE/PASP ratio was found in this study cohort (44). A retrospective analysis performed by Hoeper et al. in 108 patients diagnosed with PH-HFpEF also revealed that each 100 ng/l increase in NT-proBNP is associated with an increased risk of death (45). Guazzi et al. also demonstrated that NT-proBNP values were higher in non-survivors among PH-HFpEF and PH-HFrEF patients, as compared to survivors, although this difference was not deemed significant in the multivariate analysis of survival (46). Table 1 summarizes recent findings of BNP/NT-proBNP in PH-LHD.

**Table 1:** Collected studies of BNP/NT-proBNP in PH-LHD.

**Authors (ref #)**	**Biomarker**	**Patients (*n*)**	**Controls (*n*)**	**Primary findings**	**Correlation of BNP/NT-proBNP with hemodynamics**
Anjan et al. (41)	BNP	HFpEF (113), w/ BNP > 100 pg/ml, prospectively enrolled	HFpEF (46), w/ BNP ≤ 100 pg/ml	Normal BNP associated w/ lower age, female gender, obesity; Elevated BNP predicts CV hospitalization/death	RV wall thickness, RV size, LA/RA size, mPAP, RAP, PVR↗ Diastolic function, RV systolic function↘
Jin et al. (42)	BNP	LHD (47), PH-LHD (35), prospectively enrolled	Healthy volunteers (36)	BNP elevated in PH-LHD patients compared to LHD patients	PASP ↗
Assad et al. (25)	BNP	CpcPH (364), IpcPH (1456), retrospective analysis	PAH (564)	BNP elevated in CpcPH and IpcPH patients compared to PAH patients	N/A
Gorter et al. (43)	NT-proBNP	IpcPH-HFpEF (46), CpcPH-HFpEF (30), prospectively enrolled	Non-PH HFpEF (21)	NT-proBNP elevated in PH-HFpEF patients compared to non-PH HFpEF patients. CpcPH-HFpEF patients have elevated NT-proBNP compared to IpcPH-HFpEF patients.	TAPSE/PASP ↘
Hussain et al. (44)	NT-proBNP	PH-HFpEF (76), retrospective analysis	Non-PH HFpEF (61)	NT-proBNP elevated in PH-HFpEF patients compared to HFpEF patients	PASP ↗ TAPSE ↘
Hoeper et al. (45)	NT-proBNP	PH-HFpEF w/ DLCO <45% predicted value (52), retrospective analysis	PH-HFpEF w/ DLCO ≥ 45% predicted value (56)	Elevated NT-proBNP associated with increased risk of death in univariate model	N/A
Guazzi et al. (46)	NT-proBNP	Survivors of PH-HFpEF and PH-HFrEF (246), prospectively enrolled	Nonsurvivors of PH-HFpEF and PH-HFrEF (47)	Elevated NT-proBNP not associated with increased risk of death in multivariate model	N/A
Miller et al. (47)	NT-proBNP	SSc PH-LHD (15), SSc PAH (9), retrospective analysis w/ prospective followup	SSc (19)	NT-proBNP significantly higher at diagnosis of SSc PAH compared to SSc PH-LHD	N/A
Mazurek et al. (48)	NT-proBNP	PH-HFpEF (39), prospectively enrolled	PAH (37)	No significant difference in NT-proBNP between PH-HFpEF and PAH	N/A

*BNP, B-type natriuretic peptide; CpcPH, combined pre- and post-capillary pulmonary hypertension; CV, cardiovascular; DLCO, diffusion capacity of the lung for carbon monoxide; HFpEF, Heart failure with preserved ejection fraction; HFrEF, heart failure with reduced ejection fraction; IpcPH, isolated post-capillary pulmonary hypertension; LA, left atrium; LAD, left atrial diameter; LHD, left heart disease; LVEDP, left ventricular end-diastolic pressure; LVEF, left ventricular ejection fraction; mPAP, mean pulmonary arterial pressure; NT-proBNP, N-terminal fragment of pro-B-type natriuretic peptide; PAH, pulmonary arterial hypertension; PASP, pulmonary arterial systolic pressure; PH, pulmonary hypertension; PH-LHD, pulmonary hypertension associated with left heart disease; PVR, pulmonary vascular resistance; RA, right atrium; RAP, right atrial pressure; RV, right ventricle; RVSP, right ventricular systolic pressure; SSc, systemic sclerosis; TAPSE, tricuspid annular plane systolic excursion. ↗ indicates positive correlation with the corresponding biomarker; ↘ indicates an inverse correlation with the corresponding biomarker*.

In contrast, Miller et al. reported that NT-proBNP levels were lower in systemic sclerosis patients with early PH due to LHD than that due to PAH (47). Mazurek et al. showed that NT-proBNP levels were similar in both PH-HFpEF and PAH patients (48). The reason for these discrepancies is not immediately clear. While the levels of BNP and NT-proBNP have not yet been compared within all PH-patient populations and cannot yet be used to differentiate between pre-capillary and PH-LHD, the increase in circulating levels of these biomarkers appears to correlate well with the worsening of clinical outcomes, particularly in PH-HFpEF patients. Whether or not the monitoring of either BNP or NT-proBNP would be meaningful and effective in identifying higher risk patients or in guiding specific therapy to improve outcomes in patients with PH-LHD needs to be further investigated.

## Known Biomarkers Associated With LHD: ET-1

**Figure 2:**
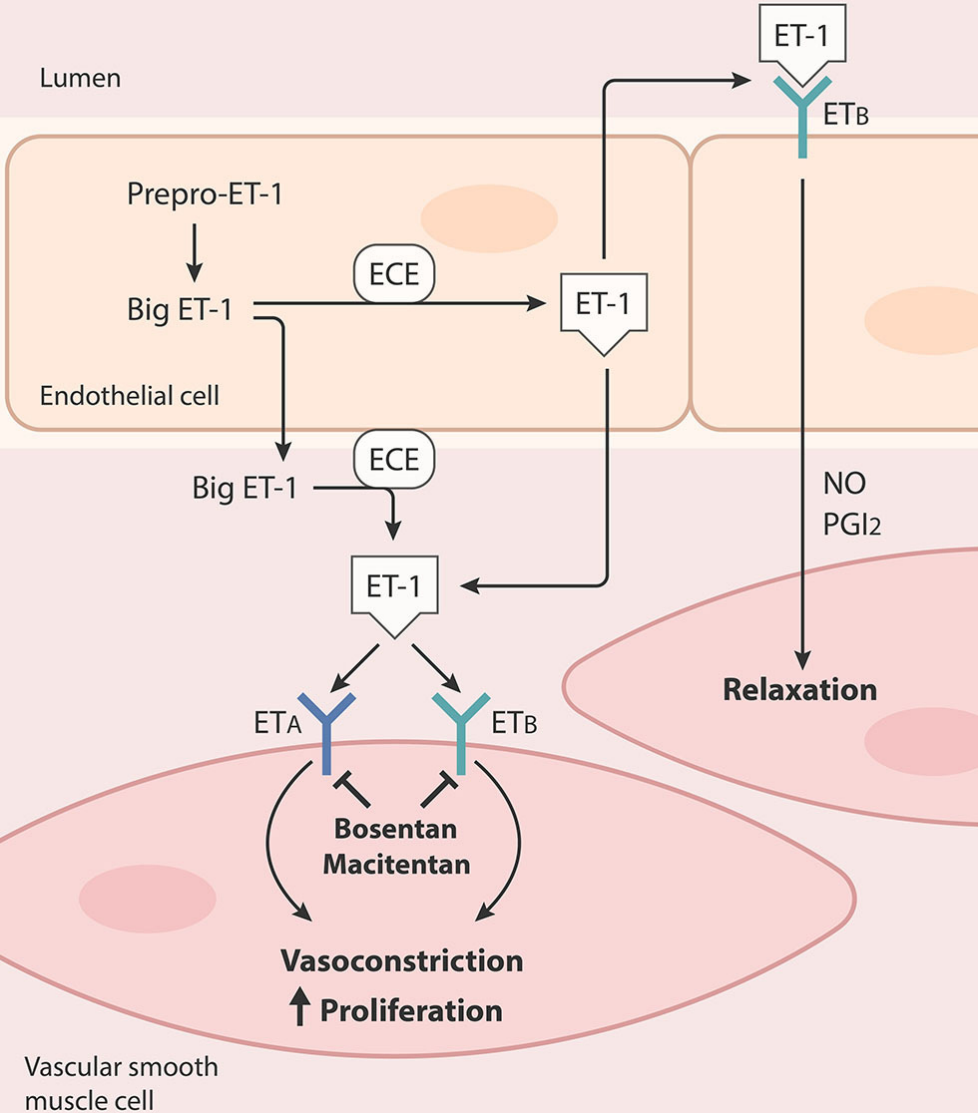
Production and release of endothelin-1 (ET-1) and ET receptors-mediated actions in vascular smooth muscle cells and endothelial cells. ET-1 is derived from prepro-ET-1, which is first proteolytically cleaved to yield a 39-amino acid intermediate Big ET-1, followed by a subsequent production of the 21-amino acid vasoactive peptide by endothelin converting enzymes (ECEs). ET-1 can active endothelin receptors type A (ET_A_) and type B (ET_B_). ET_A_ is located predominantly in vascular smooth muscle cells, while ET_B_ resides in vascular smooth muscle cells and endothelial cells. Activation of ET_A_ or ET_B_ in vascular smooth muscle cells results in vasoconstriction and proliferation. Activation of ET_B_ induces transient vasodilation in endothelial cells by releasing nitric oxide (NO) and prostacyclin (PGI_2_).

As mentioned above, chronic elevation in pulmonary capillary pressure may result in reduced pulmonary vascular compliance, structural abnormalities, and vasoconstriction in the pulmonary vasculature. Endothelial dysfunction, which leads to a reduction in NO production and an increase in ET-1 levels, is thought to be a significant contributor to these changes (13, 17, 19, 21, 22). ET-1 is the most potent endogenous vasoconstrictor known at present and has been frequently reported to be elevated in patients with HFpEF, HFrEF, and left-sided valvular disease (49–53). It is derived from prepro-ET-1, which is first proteolytically cleaved to yield a 39-amino acid intermediate Big ET-1, followed by a subsequent production of the 21-amino acid vasoactive peptide by endothelin converting enzymes (ECEs) (Figure 2) (54). The production and release of both Big ET-1 and ET-1 are promoted in response to increased myocardial stress, shear stress, low levels of estrogen, hyperglycemic conditions, oxidized LDL cholesterol, elevated proinflammatory cytokines, and other conditions that are commonly involved in the progression of LHD, though Big ET-1 has at least two orders of magnitude less vasoconstrictor potency than the mature ET-1 (55, 56). ET-1 acts at two different G protein-coupled receptors, ET_A_ and ET_B_. ET_A_ receptors are located predominantly in vascular smooth muscle cells and myocytes, and are known for their potent and long-lasting vasoconstrictive and proliferative responses to ET-1. ET_B_ activation, on the other hand, exerts vasoconstriction in smooth muscle cells but induces transient vasodilation in endothelial cells by releasing NO and prostacyclin. ET-1 levels were found to be increased in blood of HF patients (51, 52). Increased plasma levels of Big ET-1 were reported in PAH patients as well (57). Recently, Meoli et al. reported that ET-1 is higher in plasma samples collected from the wedge position of CpcPH-HFpEF patients compared to HFpEF patients with IpcPH or without PH (58). Although the sample size was small, increased wedge plasma ET-1 concentration in this study was reported to correlate strongly with PVR in patients with CpcPH-HFpEF. A slightly larger prospective cohort study performed by Chowdhury et al. showed that regardless of being associated with CpcPH or IpcPH, the wedge plasma concentration of ET-1 in PH-HFpEF patients is higher and is associated with PH, PVR, and 1-year heart failure hospitalization compared to HFpEF patients without PH (59). Similarly, Obokata et al. reported that patients with confirmed HFpEF display elevated levels of C-terminal pro-ET-1 (CT-proET-1), a stable circulating precursor of ET-1, the magnitude of which is associated with higher pulmonary artery pressure and worse pulmonary artery compliance at rest and during exercise (52). Increased plasma CT-proET-1 levels in this study also showed high diagnostic accuracy in identifying patients with abnormal pulmonary vascular reserve during exercise. Although ET-1 may play a role in the pathophysiology of PH-HFpEF, the results of using FDA-approved endothelin receptor antagonists for the treatment of PAH have been rather disappointing in the treatment of patients with PH-HFpEF. The pilot study of bosentan, a dual ET_A_ and ET_B_ receptor antagonist, showed no signs of benefit in PH-HFpEF patients (60). Moreover, this treatment may even be detrimental in the combined populations of CpcPH- and IpcPH-HFpEF patients. Bosentan demonstrated similar harmful effects during a clinical trial for the treatment of PH-HFrEF patients (61). The MELODY-1 trial, which randomized patients with CpcPH (> 75% normal ejection fraction), to a 12-week treatment with macitentan, a dual ET_A_ and ET_B_ receptor antagonist, or a placebo showed an increased incidence of side effects in the macitentan group (mainly fluid retention), without any major improvements in any of the exploratory endpoints (62). Currently, the SERENADE trial, a phase IIb clinical trial, is recruiting patients with HFpEF associated with pulmonary vascular disease or RV dysfunction to evaluate the long-term (24–52 weeks) treatment effect of macitentan (NCT03153111).

## Emerging Biomarkers: VEGF-D/FIGF

Beyond vasoconstrictive and proliferative responses mediated by ET-1, increased secretion of inflammatory cytokines and growth factors (e.g., transforming growth factor alpha 1, TGF-α1; vascular endothelial growth factor, VEGF; and interleukin 1, IL1) have also been associated with structural and functional changes in response to the retrograde increase in pulmonary capillary pressure (22). Among them, the role of vascular endothelial growth factor-D (VEGF-D) has been recently evaluated in PH-LHD. VEGF-D is a secreted factor that regulates angiogenesis, lymphangiogenesis and vascular permeability (63, 64). It is synthesized and secreted as a large precursor, which is subsequently proteolytically processed at both N- and C-termini to yield the mature forms. Unprocessed VEGF-D is selective for vascular endothelial growth factor receptor 3 (VEGFR-3), which is mainly expressed in lymphatic endothelial cells, whereas the mature VEGF-D activates both VEGFR-3 and VEGFR-2, the latter of which is found in both vascular and lymphatic endothelial cells (64). Besides modulating the growth of blood and lymphatic vessels, VEGF-D has been shown to induce cardiac fibrogenesis by stimulating myofibroblast growth, migration, and type I collagen synthesis (65). VEGF-D has also been shown to be up-regulated by mechanistic (formerly mammalian) target of rapamycin (mTOR), a master regulator of cell growth, proliferation, and survival that has been implicated in PAH, lymphangioleiomyomatosis (LAM), and cancer (66). While adenoviral delivery of a gene encoding mature VEGF-D has been shown to improve myocardial perfusion in pigs and has now advanced through phase I/IIa studies in patients with refractory angina (67, 68), elevated circulating levels of VEGF-D have been reported in patients with HF, atrial fibrillation, ischemic stroke, PAH, chronic thromboembolic pulmonary hypertension (CTEPH) and LAM (69–73). Circulating VEGF-D levels were found to be higher in HF patients with pulmonary congestion (70). Elevated VEGF-D levels were also found to be a predictor of all-cause mortality in patients with coronary artery disease (CAD) and enabled differentiation of subjects with HF from patients with acute dyspnea (70, 74). Recently, Säleby et al. reported that VEGF-D levels were elevated in patients with PAH, CTEPH, PH-HFpEF, and PH-HFrEF compared to control subjects and HF patients without PH, with its levels in PH-HFrEF patients being significantly higher compared to all other etiologies of PH (72). Of note, plasma levels of VEGF-A were higher in patients with PAH, CTEPH, and PH-LHD compared to controls, although some controversy exists regarding the presence or absence of significant differences in VEGF-A between PH etiologies (71, 72). However, data are more consistent for VEGF-D, showing increased levels in PH-LHD compared to PAH and CTEPH patients. Furthermore, levels of soluble fms-like tyrosine kinase 1 (sFlt-1, also known as soluble VEGFR-1 or sVEGFR-1) were significantly elevated in PH-LHD patients compared to controls, PAH, and no-PH LHD patients (71). These results are in agreement with a study by Houston et al., in which the authors showed that higher VEGF-D levels correlate with increased PAWP, reduced cardiac output, and higher BNP levels in patients with heart failure (75). A lower ejection fraction (30 ± 18 vs. 49 ± 22%) was also found in subjects with higher VEGF-D levels and elevated PAWP compared to those with lower VEGF-D levels in this study cohort. While there is no data available regarding the effect of therapies targeting VEGF-D in HF, PH, and PH-LHD patients, early treatment with VEGFR3 inhibitors has been shown to improve the lumen obliteration and pulmonary pressures in Sugen/hypoxia rat models of PAH (76). Collectively, the available evidence suggests that VEGF-D could be a potential biomarker for distinguishing PH-HFrEF from other etiologies of PH, but this needs to be further evaluated in large multi-center studies.

## Potential Biomarkers: microRNA-206

microRNAs (miRNAs) have been the subject of much excitement in molecular biology in the last few years. Composed of single-stranded non-coding RNAs that self-pair into a stem-and-loop structure, miRNAs act as post-transcriptional modifiers of their messenger RNA (mRNA), inducing their degradation and/or translational repression. While some controversy still exists as to their exact mechanism in identifying gene targets and roles in regulating diseases, miRNAs have emerged as promising biomarkers due to their relatively high stability (77). They can be detected extracellularly and have been implicated in a wide range of diseases. Several miRNA microarray profiling and quantitative PCR array studies have reported candidate miRNAs with potential utility as biomarkers in HF and PAH (*see* Fernandez et al., Wong et al., and Boucherat et al. for recent reviews in detail) (22, 78, 79); however, only a few miRNAs have been studied in PH-LHD. Among them, circulating levels of muscle-specific miR-204 were reported to be uniquely elevated across the pulmonary vasculature in patients with PAH, but not in patients with PH-LHD (80). On the other hand, muscle-specific miR-206 levels were found to be reduced in serum of patients with PH-HFpEF, PH-HFrEF, and valvular disease-associated PH (42). Notably, decreased miR-206 levels in this study cohort correlated with an increase in PASP, BNP, and left atrial longitudinal diameter (LAD). In addition, the authors found that the predictive value of miR-206 in detecting PH in LHD was greatly improved when combined with BNP and LAD. The exact role of miR-206 in PH-LHD is currently unknown; however, reduction of miR-206 has been shown to stabilize hypoxia-inducible factor-1α (HIF-1α) and to induce proliferation in cultured PASMCs (81, 82). In contrast, miR-206 levels were found to be elevated in lungs of rats with monocrotaline (MCT)-induced PAH, and cardiac-specific overexpression of miR-206 also led to cardiac hypertrophy (83, 84). While the reduction of circulating miR-206 levels may be compensated for by an increase in the intracellular miR-206 concentration, further research is needed to determine whether circulating miR-206 can be useful in the early identification of PH in LHD patients.

## Future Candidate Biomarkers

**Figure 3:**
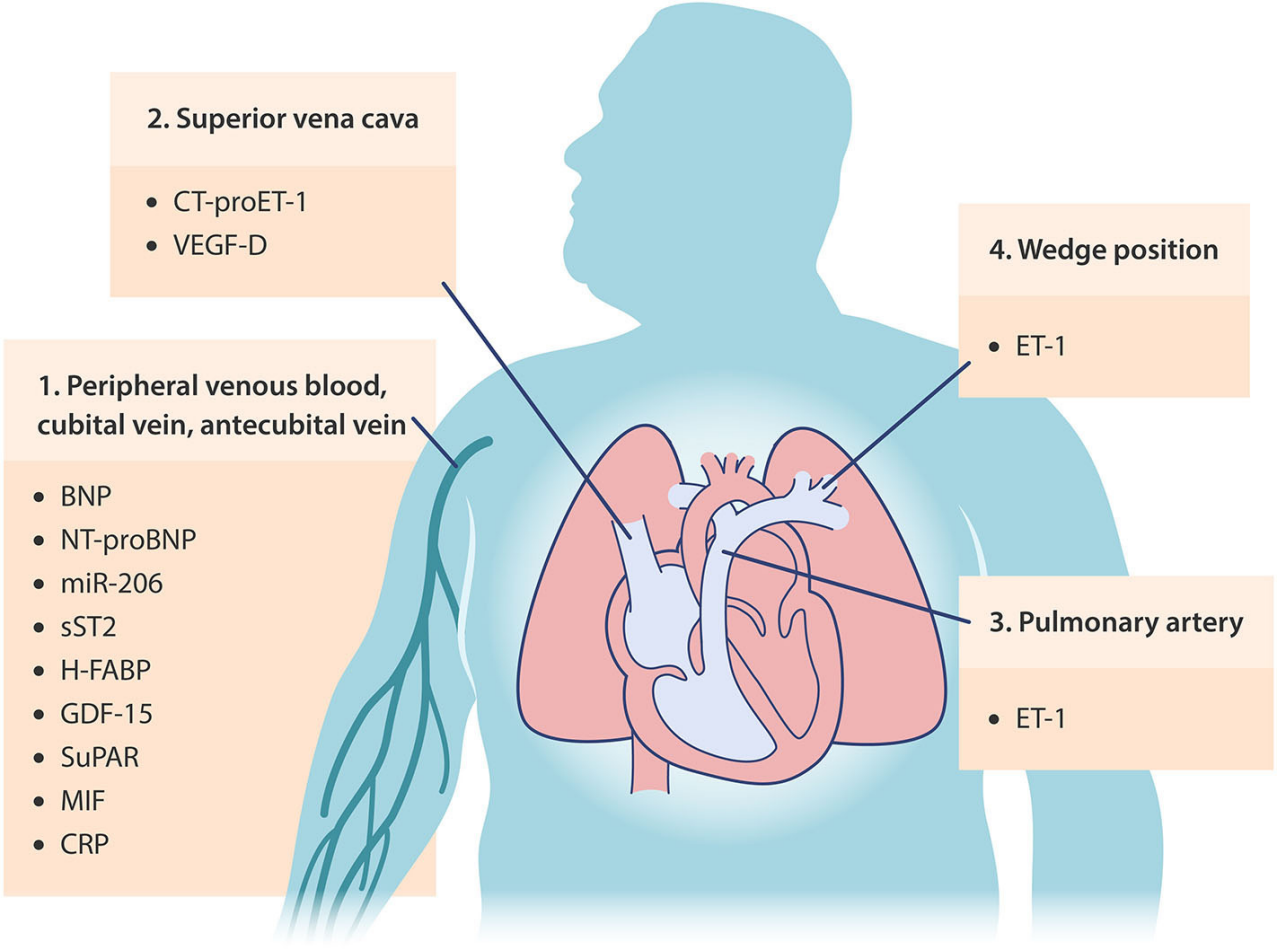
Occurrence locations of potential circulating biomarkers in PH-LHD. BNP, brain/B-type natriuretic peptide; NT-proBNP, N-terminal proBNP; miR-206, microRNA-206; sST2, soluble suppression of tumorigenicity 2; H-FABP, heart type fatty acid binding protein; GDF-15, growth differentiation factor 15; suPAR, soluble urokinase plasminogen activator receptor; MIF, macrophage migration inhibitory factor; CRP, C-reactive protein; ET-1, endothelin-1; CT-proET-1, C-terminal pro-ET-1; VEGF-D, vascular endothelial growth factor-D.

In addition to the emerging biomarkers discussed above, several biomarkers of distinct processes in cardiopulmonary regulation may aid in the characterization, differentiation, and/or determination of disease management of PH-LHD (Figure 3 and Table 2). Soluble suppression of tumorigenicity 2 (sST2), also known as interleukin-1 receptor-like 1 (IL1RL1), is one such example. sST2 is a marker of mechanical stress and ventricular remodeling, which identifies patients with HFrEF and has been shown recently to be elevated in plasma of patients with Group 2 PH, compared to subjects admitted for elective coronary angiography (in whom CAD was excluded), in a single-center retrospective study performed by Mirna et al. (85). While cautious interpretation of data is needed and detailed information regarding baseline characteristics, especially for Group 2 patients, is lacking, Mirna et al. also showed that Group 2 PH patients had higher circulating levels of growth differentiation factor 15 (GDF-15; a marker of cell injury and inflammation), heart type fatty acid binding protein (H-FABP; a marker of ongoing myocardial damage), and soluble urokinase plasminogen activator receptor (suPAR; a marker of ongoing inflammation) (85). In addition, higher plasma levels of macrophage migration inhibitory factor (MIF), a proinflammatory cytokine, were found to be associated with greater PASP and natriuretic peptide levels in HFpEF patients (86). Mazurek et al. also showed a trend toward higher levels of galectin-3, a beta-galactoside-binding protein which has been implicated in inflammation and fibrosis, in serum collected from pulmonary artery of patients with PH-HFpEF relative to those with PAH (48). While this may be associated with greater systemic fibrosis and inflammation seen in HFpEF populations, no correlations between galectin-3 levels and any measured hemodynamic endpoints were found in PH-HFpEF or PAH patients in this study (48). The common biomarker of inflammation and cardiovascular distress, C-reactive protein (CRP), has also gained some interest as a potential biomarker of PH-LHD. Using a CRP cutoff value of > 3 mg/dl, the RELAX trial reported that approximately 60% enrolled HFpEF patients have elevated CRP (87). Higher CRP levels were associated with younger age, higher BMI, COPD, atrial fibrillation, RV dysfunction, reduced exercise tolerance, higher circulating levels of ET-1, aldosterone, and NT-pro BNP. While this led the authors to conclude that high CRP levels may identify a unique HFpEF phenotype that is associated with comorbidity-driven systemic inflammation, CRP levels were not associated with PH or LV function in this study cohort (87). Lastly, platelets from patients with PH-HFpEF have also been shown to exhibit a distinct metabolic phenotype compared to patients with PAH (88). Unlike platelets from PAH patients, only an increase in maximal respiratory capacity, but not in glycolytic rate, was observed in platelets from PH-HFpEF patients relative to healthy controls. The enhancement of platelet maximal respiratory capacity was found to be associated with RV dysfunction, but not with mPAP or PVR in patients with PH-HFpEF (88). Whether or not a measurement of these factors can provide a method for distinguishing PH-LHD patient phenotypes, identifying at-risk subjects, and predicting PH-LHD patient outcomes requires additional research.

**Table 2:** Collected studies of future candidate biomarkers in PH-LHD.

**Authors (ref #)**	**Biomarkers**	**Patients (*n*)**	**Controls (n)**	**Primary findings**	**Correlation with hemodynamics**
Mirna et al. (85)	sST2, GDF-15, H-FABP, suPAR, BNP	Group 1 PH (13), Group 2 PH (35), Group 3 PH (7), Group 4 PH (12), Group 5 PH (19), retrospective analysis	Elective coronary angiography w/o CAD (74)	BNP, GDF-15, H-FABP, and suPAR are elevated in Group 2 PH patients and correlate with some hemodynamic measurements	H-FABP w/ RVEDD, RAA, TR: ↗ sST2 w/ RVEDD, RAA, TR, PASP: ↗ sST2 w/ TAPSE: ↘ GDF-15 w/ RVA, RAA: ↗ suPAR w/ RVEDD, RAA, TR: ↗ suPAR w/ TAPSE: ↘
Luedike et al. (86)	MIF	HFpEF w/ MIF > 51.58 ng/ml (31), prospectively enrolled	HFpEF w/ MIF ≤ 51.58 ng/ml (31)	High MIF associated w/ 180-day mortality/hospitalization and more severe HFpEF symptoms	MIF w/ PASP: ↗
Mazurek et al. (48)	Galectin-3	PH-HFpEF (39), prospectively enrolled	PAH (37)	Galectin-3 positively correlated with mortality	N/A
DuBrock et al. (87)	hs-CRP	HFpEF w/ CRP > 3 mg/l (121), retrospective analysis	HFpEF w/ CRP ≤ 3 mg/l (93)	High CRP associated with increased comorbidity burden and presence of RV dysfunction	N/A
Nguyen et al. (88)	Platelet bioenergetics	PH-HFpEF (20), retrospective analysis	Healthy volunteers (20)	PH-HFpEF patient platelets display increased maximal OCR and reserve respiratory capacity	Reserve respiratory capacity w/ RV SWI: ↘

*CAD, coronary artery disease; GDF-15, growth differentiation factor 15; H-FABP, heart-type fatty acid binding protein; hs-CRP, high-sensitivity C-reactive protein; MIF, macrophage inhibitory factor; OCR, platelet oxygen consumption rate; RAA, right atrial area; RVEDD, right ventricular end diastolic diameter; sST2, soluble suppression of tumorigenicity 2; suPAR, soluble urokinase plasminogen activator receptor; SWI, stroke work index; TR, severity of tricuspid regurgitation. ↗ indicates positive correlation with the corresponding biomarker; ↘ indicates an inverse correlation with the corresponding biomarker*.

Apart from clinical studies, recent advances in pre-clinical models have provided new insights in the development of future biomarkers. Plasma levels of leptin, an adipokine known to induce secretion of proinflammatory cytokines and reactive oxygen species (ROS) generation, were found to be associated with PH severity in rats with PH-HFpEF induced by high-fat diet (HFD) in combination with supra-coronary aortic banding (SAB) and the antipsychotic olanzapine (89). Decreased plasma levels of the cardioprotective adipokine adiponectin after Sugen-mediated induction of PH-HFpEF were also observed in leptin receptor-deficient obese ZSF1 rats (90). Interestingly, metformin (the first-line drug for type 2 diabetes) was found to improve metabolic syndrome and pulmonary pressures in these models through modulation of leptin, adiponectin, as well as other mechanisms involving, at least in part, suppression of interleukin-6-associated inflammation and activation of sirtuin-3-mediated skeletal muscle glucose uptake (89, 90). Moreover, metformin has recently been shown to prevent RV dysfunction via improved insulin resistance and reduced RV lipid accumulation in HFD-treated mice (91). These findings have led to an ongoing trial designed to evaluate the effect of metformin in patients with PH-HFpEF (NCT03629340). Nitrite, a dietary precursor of NO, has displayed similar qualities in a rat model of PH-HFpEF where it functions to increase adiponectin levels and improve skeletal muscle insulin resistance (90). A recent study by Simon et al. reported that inhalation of nitrite reduced pulmonary, RA, and pulmonary capillary wedge pressures in patients with PH-HFpEF (92). A clinical trial is now underway to examine the effect of oral nitrite in PH-HFpEF patients (NCT03015402). Moreover, therapies targeting ET-1 and NO with a Rho-kinase (ROCK) inhibitor, fasudil, have also been shown to be effective in rats with end-stage PH-LHD induced by SAB and in patients with PH-HFpEF, with more favorable results in the CpcPH-HFpEF population (93, 94).

## Summary and Future Perspectives

While many challenges still exist, researchers in the field are encouraged to further expand the growing array of circulating biomarkers to guide future research toward facilitating screening, diagnosis, refinement of specific patient phenotypes, and development of therapeutic approaches. Consensus to concise and standardized definition for hemodynamic phenotyping is required, along with longitudinal data consisting of multiple time points and evaluation in large, multi-center studies to validate the viability of the aforementioned potential biomarkers and to explore future candidates. The ongoing PVDOMICS study supported by the National Institutes of Health/National Heart, Lung, and Blood Institute (NIH/NHLBI) may also reveal new relevant information (95). Since HFpEF and PH are recognized as multiorgan and systemic disorders (96, 97), the quest toward identification of meaningful extra-cardiopulmonary biomarkers may aid in providing important new insights into this clinically challenging syndrome and driving PH-LHD research forward.

## Author Contributions

NT and Y-CL wrote the article.

## Conflict of Interest

The authors declare that the research was conducted in the absence of any commercial or financial relationships that could be construed as a potential conflict of interest.
